# Effect of venous foot pump intervention on prevention of venous thromboembolism in patients with major orthopedic surgery: a systematic review and meta-analysis

**DOI:** 10.3389/fcvm.2024.1408334

**Published:** 2024-08-02

**Authors:** Yahui Tong, Rulan Ying, Meier Niu, Lan Xu

**Affiliations:** Department of Nursing, The First Affiliated Hospital of Soochow University, Suzhou, China

**Keywords:** venous foot pump, major orthopedic surgery, venous thromboembolism, systematic review, meta-analysis

## Abstract

**Background:**

Venous thromboembolism (VTE) is a common complication after major orthopedic surgery. The venous foot pump (VFP) is an effective mechanical preventive measure against VTE in patients. However, the differences in effectiveness based on varying usage times of VFP remain unclear.

**Objective:**

To explore the effectiveness of VFP with different usage times in preventing VTE in patients undergoing major orthopedic surgery.

**Methods:**

Nine databases (PubMed, Web of Science, CINAHL, Embase, Cochrane Library, CBM, VIP, CNKI, and Wanfang) were searched to identify randomized controlled trials (RCTs) evaluating VFP interventions for VTE prevention in major orthopedic surgery patients. The risk of bias in each study was assessed using the Cochrane Collaboration tool. Meta-analysis was conducted using RevMan 5.3.

**Results:**

A total of 36 RCTs involving 3,791 patients undergoing major orthopedic surgery were included. Meta-analysis revealed significant differences in VTE incidence between the VFP and blank control groups (RR = 0.27, 95% confidence interval CI: 0.19–0.38, *P* < 0.001) and between the VFP plus chemoprophylaxis and chemoprophylaxis alone groups (RR 0.39, 95% CI: 0.29–0.53, *P* < 0.001). However, no statistically significant difference was observed between the VFP and the LMWH groups (RR = 0.93, 95% CI: 0.54–1.61, *P* = 0.8). Subgroup analysis showed no significant difference in effectiveness based on different VFP usage durations (VFP vs. Blank: Chi-square = 0.54, *P* = 0.46, I^2^ = 0%; VFP Plus chemoprophylaxis vs. chemoprophylaxis alone: Chi-square^ ^= 1.93, *P* = 0.86, I^2 ^= 0%).

**Conclusion:**

The current evidence indicates that VFP significantly reduces the incidence of postoperative VTE in patients undergoing major orthopedic surgery. VFP can be considered an add-on strategy to LMWH for patients at low risk of bleeding and an alternative strategy to LMWH in patients at high risk of bleeding. This study found no significant difference in effectiveness between various VFP usage interventions. Future research should focus on economic cost-effectiveness and patient acceptance to help policymakers determine the most efficient usage duration, providing practical guidance for thromboprophylaxis.

## 1 Introduction

Venous thromboembolism (VTE) contains deep vein thrombosis (DVT) and pulmonary thromboembolism (PTE). DVT refers to abnormal blood clotting in the deep veins, usually in the lower limbs (1), leading to potential dysfunction or disability. PTE occurs when a blood clot dislodges and travels through the bloodstream to the pulmonary artery. According to risk assessments for VTE in orthopedic surgery patients (2), those undergoing major orthopedic surgeries, such as hip arthroplasty (HA), knee arthroplasty (KA), and hip fracture surgery (HFS) (3), are at high risk for VTE. Studies (4) have revealed that without preventive measures, the incidence of DVT after total HA ranges from 20.6% to 47.1% and from 30.8% to 58.2% after total KA. VTE often lacks typical clinical symptoms but significantly impacts patients’ quality of life and can result in perioperative mortality (2). Therefore, early screening and prevention are crucial in VTE prevention (5, 6).

The guidelines (2) strongly recommend implementing effective preventive measures in patients undergoing major orthopedic surgery to reduce the incidence and mortality of VTE, alleviate patient suffering, and reduce healthcare costs. Current preventive measures for VTE in major orthopedic patients mainly include physical and drug prevention and basic preventive measures. Physical prevention is an essential strategy, serving as a necessary supplement and alternative to drug prevention in specific situations. The combination of physical and drug prevention addresses multiple aspects of Virchow's triad, thus synergistically reducing the occurrence of VTE (7). Drug prevention primarily involves using anticoagulant drugs such as LMWH, Xa factor inhibitors, and vitamin K antagonists, which can inhibit the coagulation process and reduce blood hypercoagulability. Physical prevention measures use pressure to accelerate blood flow in the lower limbs, reducing blood stasis and significantly reducing the occurrence of postoperative lower limb DVT while minimizing bleeding risks. The guidelines for the prevention and treatment of VTE recommend physical prevention methods for thrombosis prevention in patients categorized as moderate and high risk for VTE, highlighting the generalizability and importance of physical prevention.

Similarly, venous foot pump (VFP) is a common mechanical precaution, and compared to graduated compression stockings (GCS) and intermittent pneumatic compression devices (IPCD), it offers the advantages of better effectiveness and ease of wear (8–13). While research on the effectiveness of VFP in preventing VTE in major orthopedic surgery patients continues to be published, there remains some controversy over the conclusions. Numerous studies have demonstrated that VFP can effectively prevent VTE in orthopedic surgery patients (14–16). However, Pitto and colleagues (17, 18) have revealed that compared to Low molecular weight heparin (LMWH), VFP does not significantly differ in preventing postoperative VTE in these patients. Moreover, the differences in the effects of VFP intervention at different usage times remain unclear, and researchers hold varying views on the optimal duration of VFP use (14, 16, 19, 20). For example, the consensus among experts in the mechanical prevention of VTE (9) suggests that IPCD should be used for at least 18 h per day. For patients who are completely immobilized, the daily usage time should be extended as much as possible. However, the usage time of VFP is not specified. Windish and colleagues (17, 19) demonstrated that continuous VFP application for 24 h can reduce the incidence of postoperative VTE in orthopedic surgery patients. Conversely, several studies (16, 20, 21) have used VFP intervention at a usage time of 30 min twice a day, showing an effective reduction of postoperative VTE in these patients. Based on the current studies, the effectiveness of VFP in postoperative VTE prevention in patients undergoing major orthopedic surgery and the optimal usage time of intervention remains to be further examined.

Therefore, this study used a systematic evaluation method to assess the effectiveness of VFP in preventing postoperative VTE in orthopedic surgery patients. It also compared and analyzed studies on VFP intervention at different usage times to provide a reference basis for clinical decision-making.

## 2 Methods

This study followed the Preferred Reporting Items for Systematic reviews and Meta-Analyses (PRISMA) statement. The PRISMA checklist is shown in Supplementary File 1 (22).

### 2.1 Search strategy

The following electronic databases were systematically searched from their inception until September 2023: PubMed, Embase, CINAHL, Cochrane Library, Web of Science, and four Chinese databases (CBM, CNKI, VIP, and WanFang). Searches in each database were performed using a combination of subject terms and free terms. The search terms included VTE (“venous thromboembolism,” “venous thrombosis,” “phlebothrombosis,” “deep venous thrombosis,” “DVT”), major orthopedic surgery (“arthroplasty,” “replacement,” “hip fracture surgery,” “orthopedic/orthopaedic,” “fracture,” “joint prosthesis implantation,” “orthopedic surgery”), VFP (“venous foot pump,” “A-V impulse system”), and RCT (“randomized controlled trial,” “randomized”). The complete search strategy for the PubMed database is provided in the Supplementary File 2).

### 2.2 Inclusion and exclusion criteria

The inclusion criteria for the literature were as follows: (1) Study population (P): patients undergoing major orthopedic surgery (including hip/knee arthroplasty and surgeries for hip fractures such as intertrochanteric fractures, subtrochanteric fractures, femoral neck fractures, femoral head fractures, and acetabular fractures); (2) intervention (I): the experimental group receiving VFP therapy; (3) control (C): the group receiving only anticoagulant therapy or serving as the blank control group; (4) outcome measures (O): incidence of DVT/PTE; (5) study type (S): randomized controlled trials (RCTs).

The exclusion criteria for the literature were as follows: (1) Studies with duplicate outcomes; (2) studies with incomplete or unextractable data; (3) studies with low quality.

### 2.3 Study selection and data extraction

Two researchers independently screened the literature based on the inclusion and exclusion criteria and extracted data from articles that met the inclusion criteria. The extracted data primarily included the first author, country, publication year, sample size, gender and age of the study subjects, intervention measures (intervention content and usage time), and outcome indicators. In case of any disagreements, they consulted a third researcher to reach the final decision.

### 2.4 Quality appraisal

The two researchers used the Cochrane risk of bias assessment tool (23) to evaluate the quality in seven aspects: random sequence generation, allocation concealment, blinding of participants and personnel, blinding of outcome assessors, completeness of outcome data, selective outcome reporting, and other biases. Each aspect was evaluated as low risk of bias, unclear risk of bias, or high risk of bias.

### 2.5 Data analysis

Data analysis was performed using the RevMan 5.3 software provided by the Cochrane Library. The relative risk (RR) and a 95% confidence interval (CI) from different studies were synthesized. Heterogeneity among the included studies was assessed using the Cochrane Q test and the calculation of the I^2^ value. If there was no statistically significant heterogeneity among the studies (I^2^ ≤ 50%, *P* ≥ 0.10), a fixed-effects model was selected for the meta-analysis. Otherwise, a random-effects model was used. If there was statistically significant heterogeneity (I^2^ > 50%, *P* < 0.10), subgroup analysis and sensitivity analysis will be used to explore the potential sources of heterogeneity. Publication bias was assessed using a funnel plot and Egger's test for studies with a sample size of 10 or more (24). A *p*-value of less than 0.05 was considered significant.

## 3 Results

### 3.1 Search outcomes

**Figure 1:**
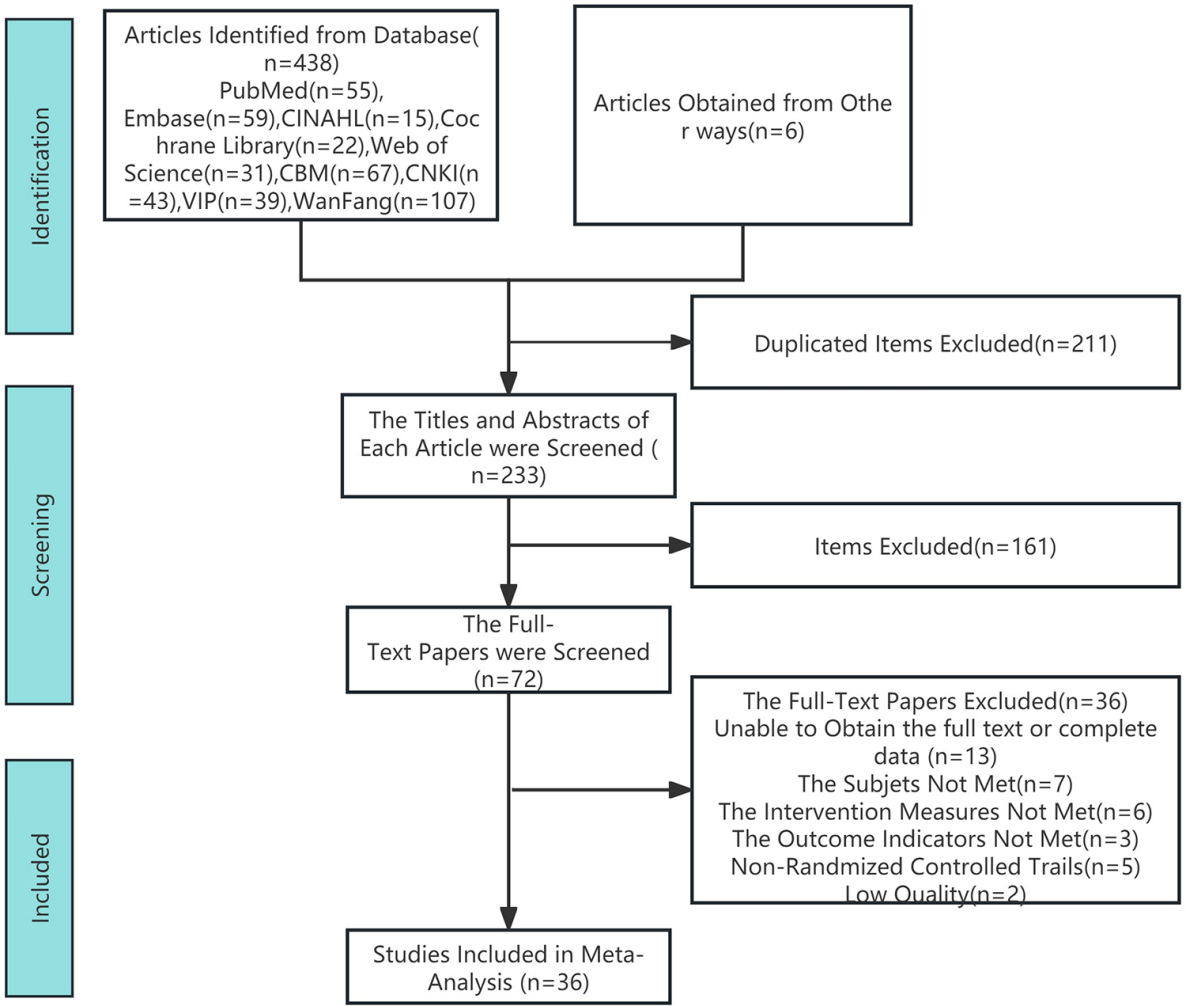
Literature screening process.

After reviewing the full texts, 36 articles were included in the final analysis (Figure 1).

### 3.2 Characteristics of included studies

**Figure 2:**
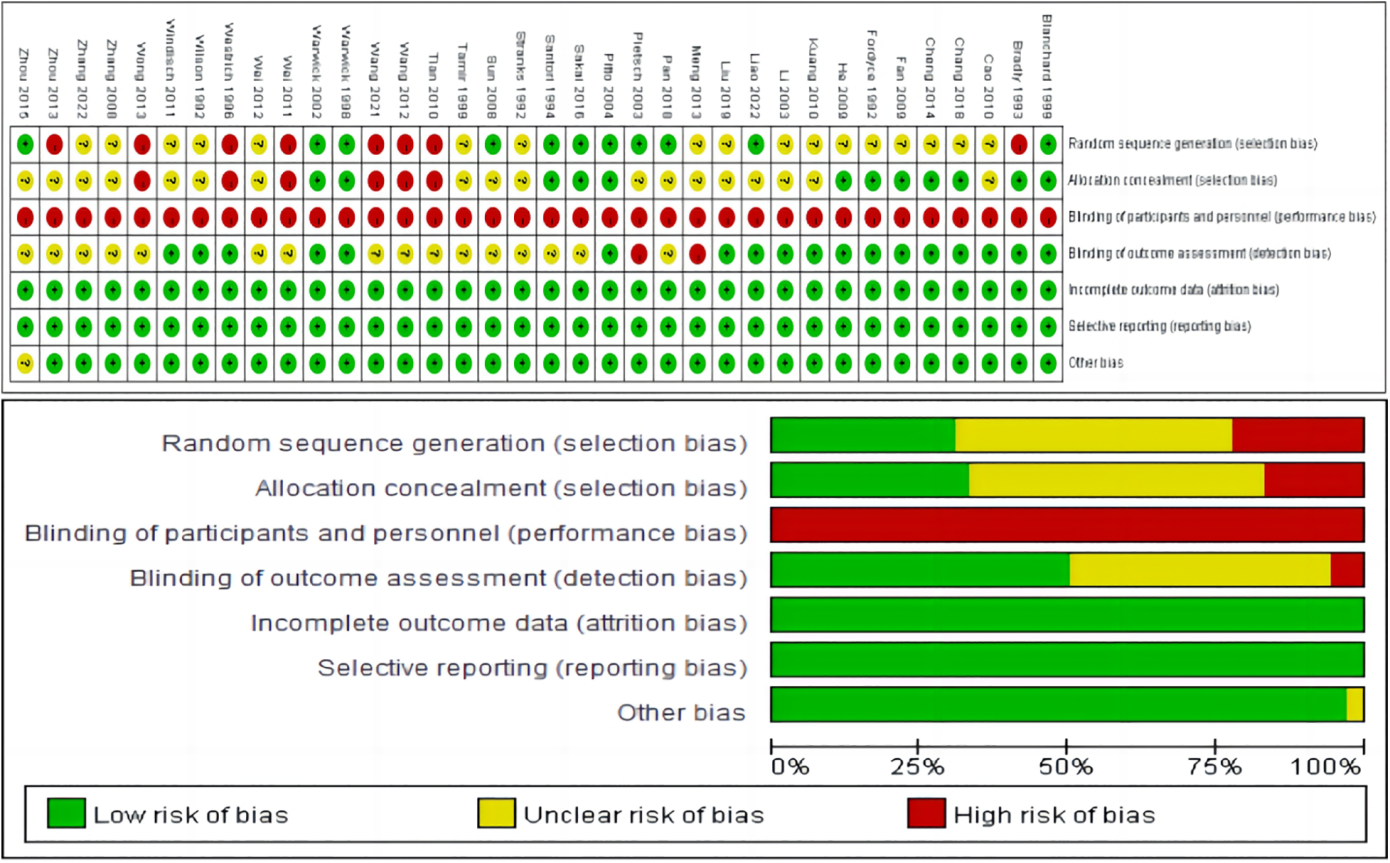
Risk bias assessment.

The basic characteristics of the 36 included articles are shown in Table 1. A total of 3,791 patients undergoing major orthopedic surgery were included in the studies. The methodological quality and risk of bias assessment of the included studies are displayed in Figure 2.

**Table 1:** The characteristics of included studies (*n* = 36).

Author	Year	Country	Sample (*n*)		Sex (*n*)		Age (median, year)		Intervention		Frequency of VFP	Outcome Indicator
			Experimental	Control	Male	Female	Experimental	Control	Experimental	Control		
Liao Z	2022	China	57	58	58	57	70.75	70.19	VFP	Blank control	VFP:20 min BID	DVT
Li HX	2003	China	42	20	55	7	52.5	50.5	VFP	Blank control	VFP:30 min BID	DVT
Pan LX	2018	China	43	43	56	30	76.2	77.9	VFP	Blank control	VFP:30 min Q8H	DVT
Fan XC	2009	China	33	33	50	16	—	—	VFP	Blank control	VFP:2 h BID	DVT
Wang LX	2012	China	100	100	109	91	69.42	66.56	VFP	Blank control	VFP:3 h BID	DVT
He LY	2009	China	28	21	21	28	69.4	66.5	VFP	Blank control	VFP:3 h BID	DVT
Sun YQ	2008	China	30	28	25	33	70	67	VFP	Blank control	VFP:24 h	DVT
Wong YC	2013	China	47	44	29	62	68.4	67.8	VFP	Blank control	VFP:24 h	DVT
Fordyce	1992	England	39	40	30	49	68.1	71.2	VFP	blank control	VFP:24 h	DVT
Stranks	1992	England	41	39	15	65	79.1	82	VFP	Blank control	VFP:24 h	DVT
Wilson	1992	England	28	32	15	44	71.1	70.1	VFP	Blank control	VFP:24 h	DVT
Blanchard	1999	Switzerland	63	67	31	99	72	74	VFP	LMWH	VFP:24 h	DVT/PE
Pietsch	2003	NewZealand	50	50	33	67	56	56	VFP	LMWH	VFP:24 h	DVT/PE
Pitto	2004	New Zealand	100	100	62	138	57.3	58.1	VFP	LMWH	VFP:24 h	DVT/PE
Santori	1994	Italy	67	65	34	98	72.4	69.8	VFP	LMWH	VFP:24 h	DVT/PE
Warwick	1998	United Kingdom	147	143	181	109	68	68	VFP	LMWH	VFP:24 h	DVT/PE
Warwick	2002	England	99	89	80	149	73	71	VFP	LMWH	VFP:24 h	DVT/PE
Wang HM	2021	China	51	51	71	31	54.71	54.37	VFP, LMWH	LMWH	VFP:20 min QD	DVT
Chang B	2018	China	64	56	58	62	71.4	73.3	VFP, LMWH	LMWH	VFP:20 min BID	DVT
Wei W	2012	China	31	31	37	25	—	—	VFP, LMWH	LMWH	VFP:20 min BID	DVT
Zhang L	2022	China	60	60	66	54	47.12	46.92	VFP, Rivaroxaban	Rivaroxaban	VFP:30 min BID	DVT
Liu ZQ	2019	China	40	40	51	29	65.12	65.11	VFP, LMWH	LMWH	VFP:30 min BID	DVT
Meng XM	2013	China	30	30	28	32	—	—	VFP, LMWH	LMWH	VFP:30 min BID	DVT
Wei L	2011	China	51	49	74	26	45.71	48.36	VFP, LMWH	LMWH	VFP:30 min BID	DVT
Cao J	2010	China	38	38	25	51	—	—	VFP, LMWH	LMWH	VFP:30 min BID	DVT
Zhang SH	2008	China	50	50	28	72	62.2	60.9	VFP, LMWH	LMWH	VFP:30 min BID	DVT
Zhou C	2015	China	40	40	57	23	—	—	VFP, aspirin	Aspirin	VFP:30 min Q8H	DVT
Kuang WB	2010	China	65	65	47	83	76.3	79.5	VFP, aspirin, warfarin	Aspirin, warfarin	VFP:30 min Q8H	DVT
Tian LZ	2010	China	56	56	47	65	—	—	VFP, LMWH, aspirin	LMWH, aspirin	VFP:2 h BID	DVT
Tamir	1999	Israel	24	24	12	36	69	70	VFP, LMWH	LMWH	VFP:16 h QD	DVT
Windisch	2011	Germany	40	40	—	—	68.93	68.15	VFP, LMWH	LMWH	VFP:24 h	DVT
Westrich	1996	New YorK	61	61	40	82	—	—	VFP, aspirin	Aspirin	VFP:24 h	DVT
Cheng P	2014	China	67	65	34	98	72.4	69.8	VFP, LMWH	VFP, LMWH	Unreported	DVT
Zhou FL	2013	China	44	43	32	55	70.09	66.15	VFP, Fondaparinux Sodium	Fondaparinux Sodium	Unreported	DVT
Sakai	2016	Japan	58	62	20	100	73	74.3	VFP, Edoxaban	Edoxaban	Unreported	DVT
Bradly	1993	United Kingdom	30	44	—	—	—	—	VFP, heparin , Hydroxychloroquine sulfate	Heparin, hydroxychloroquine sulfate	Unreported	DVT

### 3.3 Meta-analysis results

#### 3.3.1 Comparison of the incidence of DVT in patients with VFP alone group and blank control group

**Figure 3:**
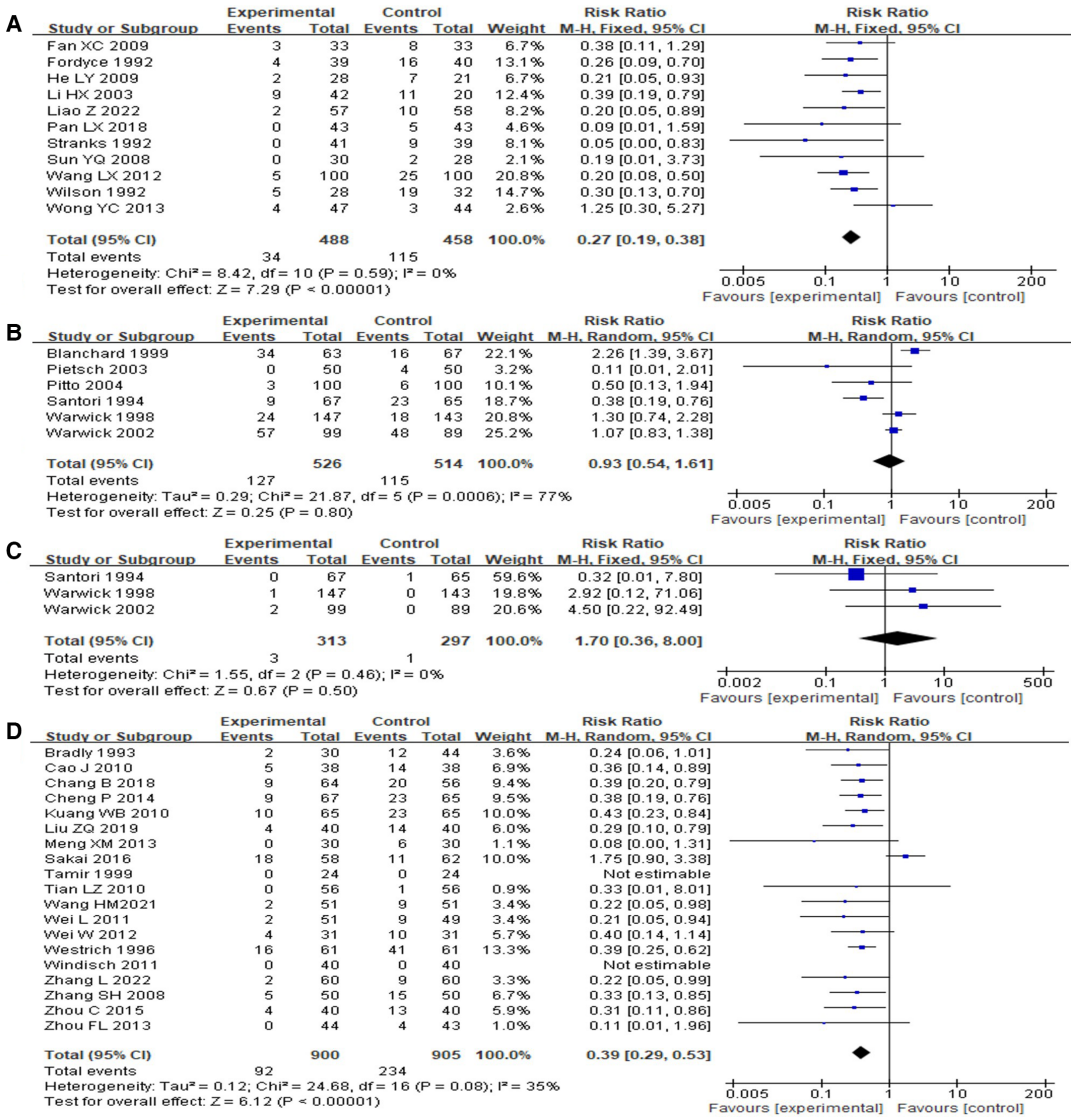
Forest map of VTE prevention effect between VFP group and control group [(**A**) VFP versus blank control; (**B**) VFP versus LMWH (outcome: DVT); (**C**) VFP versus LMWH (outcome: PTE); (**D**) VFP plus chemoprophylaxis versus chemoprophylaxis alone].

Eleven studies (15, 25–34) compared the DVT incidence between VFP and blank control group. The results depicted a significant difference in the incidence of postoperative DVT between the two groups of orthopedic surgery patients (pooled RR = 0.27, 95% CI: 0.19–0.38, *P* < 0.001; I^2 ^= 0%, *P* = 0.59) (Figure 3A).

**Figure 4:**
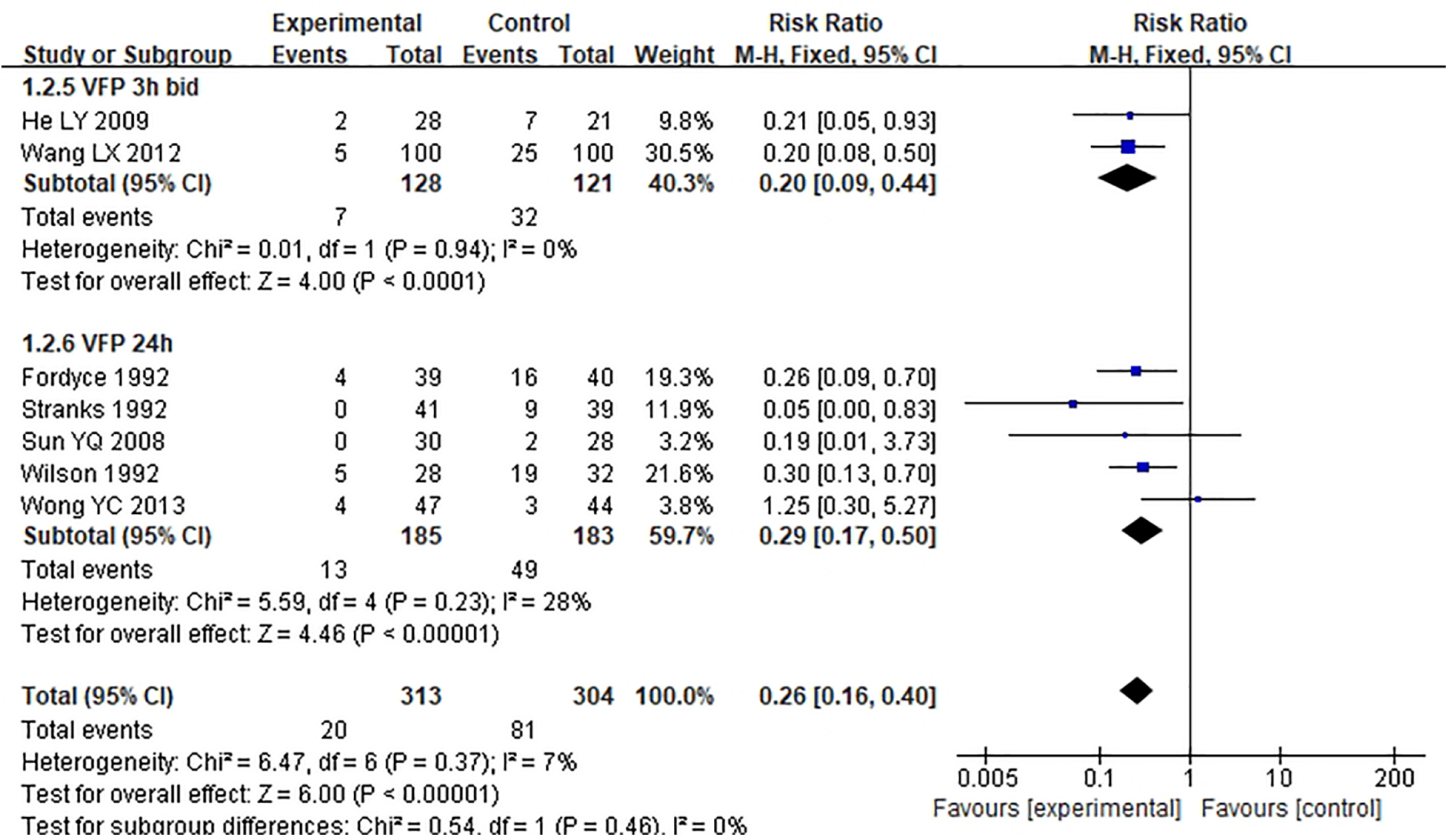
Subgroup analysis of different frequencies of VFP alone.

Among the 11 studies, there were 2 different VFP intervention usage times (3 h BID, 24 h). Subgroup analysis of the two usage times showed that the RR values were 0.20 (3 h BID), 0.29 (24 h) in order from low to high. However, there was no statistical significance among the subgroups (Chi-square^ ^= 0.54, *P* = 0.46, I^2 ^= 0%) (Figure 4).

#### 3.3.2 Comparison of the incidence of DVT in patients with VFP group and LMWH group

Six studies (17, 18, 35–38) compared the incidence of DVT between the VFP group (24 h) and the LMWH group. The results indicated a non-significant difference in the incidence of postoperative DVT between the VFP group and the LMWH group in orthopedic surgery patients (RR = 0.93, 95% CI: 0.54–1.61, *P* = 0.8; I^2 ^= 77%, *P* = 0.0006) (Figure 3B).

Three of the six studies (36–38) compared the incidence of PTE between the VFP group (24 h) and the LMWH group. The results revealed a non-significant difference in the incidence of postoperative PTE between the VFP group and the LMWH group in orthopedic surgery patients (pooled RR = 1.7, 95% CI: 0.36–8.00, *P* = 0.5; I^2 ^= 0%, *P* = 0.46) (Figure 3C).

#### 3.3.3 Comparison of the incidence of DVT in patients with VFP plus chemoprophylaxis group and chemoprophylaxis alone group

A total of 19 studies (14, 16, 19–21, 39–52) were included in the meta-analysis comparing the incidence of DVT between the group receiving VFP combined with anticoagulant drugs and the group receiving anticoagulant drugs alone. The results showed a significant difference in the incidence of postoperative DVT between the two groups of orthopedic patients [RR = 0.39, 95% CI (0.29, 0.53), *P* < 0.001; I^2 ^= 35%, *P* = 0.08] (Figure 3D).

**Figure 5:**
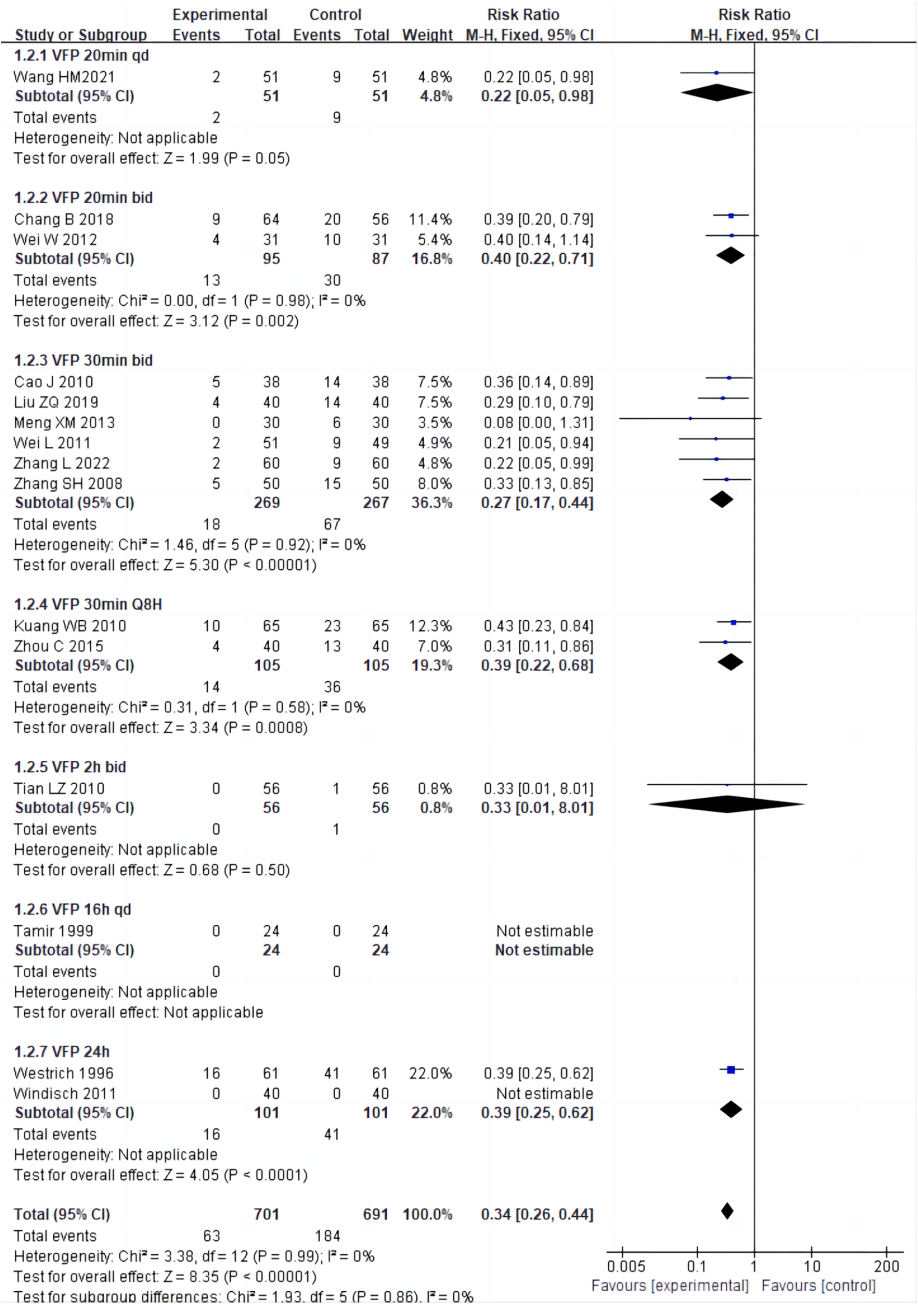
Subgroup analysis of different frequencies of VFP plus chemoprophylaxis.

Among the 15 studies (16, 19–21, 40, 41, 43–51), there were 7 different usage times of VFP intervention (20 min once daily, 20 min twice daily, 30 min twice daily, 30 min every eight hours, two hours twice daily, 16 h once daily, 24 h). Subgroup analysis of the seven usage times showed that the RR values were 0.22 (20 min QD), 0.27 (30 min BID), 0.33 (2 h BID), 0.39 (30 min Q8 H), 0.39 (24 h), 0.40 (20 min BID) and “not estimable” (16 h QD). However, there was a non-significant difference among the subgroups (Chi-square^ ^= 1.93, *P* = 0.86, I^2 ^= 0%) (Figure 5).

#### 3.3.4 Sensitivity analysis and publication bias

**Figure 6:**
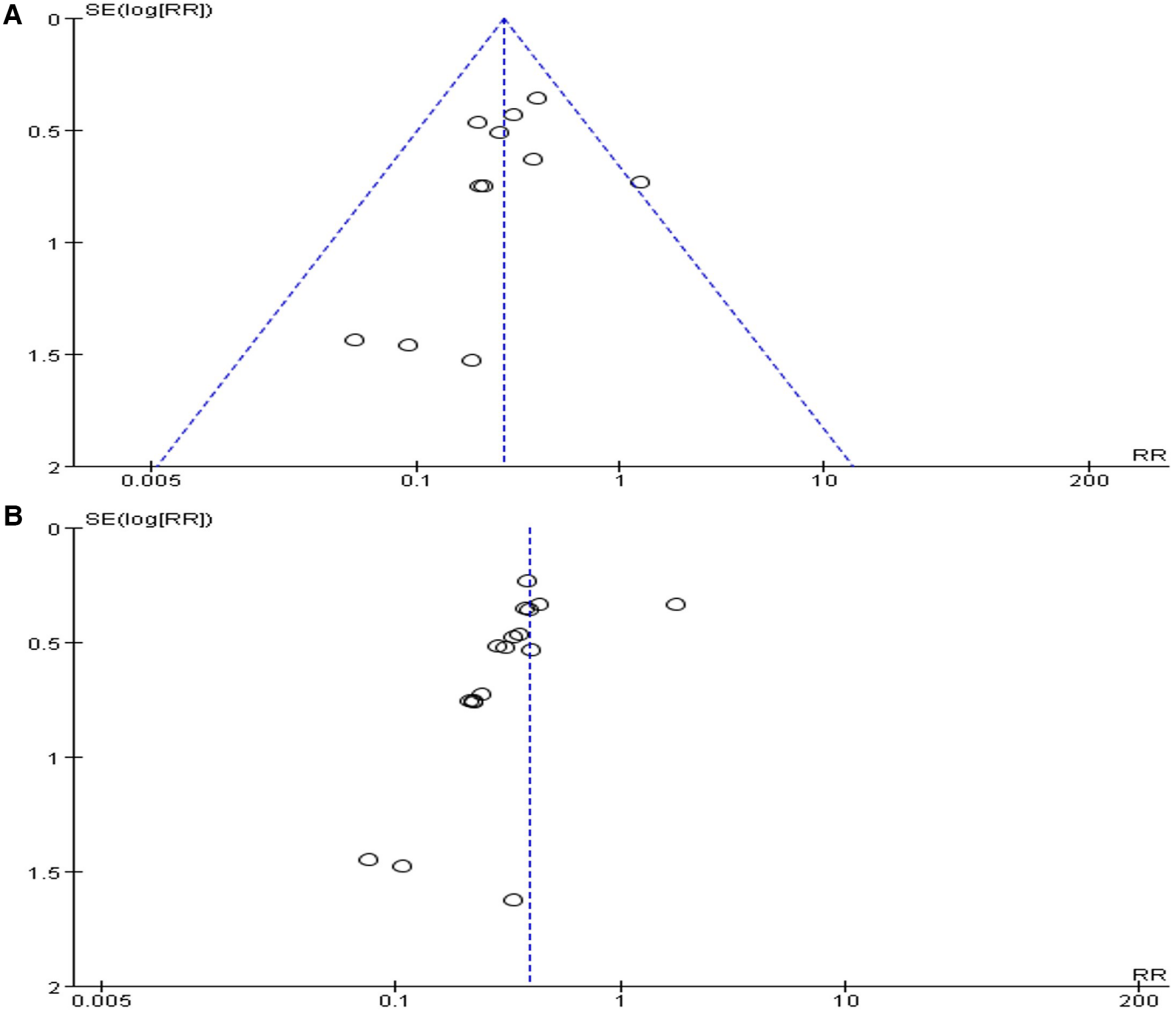
Funnel plot [(**A)** VFP versus blank control; (**B**) VFP plus chemoprophylaxis versus chemoprophylaxis alone].

Sensitivity analysis disclosed that the results were robust. Funnel plot and Egger's test were performed to analyze publication bias for the 11 studies comparing the VFP group with the blank control group and the 19 studies comparing the VFP combined with the anticoagulant drugs group with the anticoagulant drugs only group. The results were non-significant in publication bias for the VFP vs. control group (t = –1.21, *P* = 0.258, *P* > 0.05) and the VFP combined with anticoagulant drugs vs. anticoagulant drugs only group (t = –2.02, *P* = 0.062, *P* > 0.05) (Figure 6).

## 4 Discussion

### 4.1 Effectiveness of VFP

The meta-analysis results of this study indicated that VFP has significantly prevented postoperative VTE in patients undergoing major orthopedic surgery. This finding is consistent with Zhang and colleagues (53) and emphasizes the critical role of VFP in reducing postoperative VTE risk in orthopedic surgery patients. VFP stimulates the body's “physiological foot pump” and generates high-speed pulsatile blood flow similar to cardiac function. This mechanism rapidly impacts the plantar region, creating a pulsatile acceleration state similar to walking. It increases blood flow velocity, prevents clotting, and avoids blood stasis and thrombus formation in the vascular endothelium. Given its low risk of bleeding, VFP can be an effective alternative to pharmacological prevention (54).

### 4.2 Effect of combined use of VFP and anticoagulant drugs

The research findings highlighted that combining VFP with anticoagulant drugs provides better prevention of postoperative DVT in orthopedic surgery. VFP not only promotes blood flow in the lower extremities but also stimulates the synthesis and release of plasminogen activators in endothelial cells, activating plasmin and facilitating fibrinolysis, preventing blood coagulation (55). Anticoagulant drugs such as rivaroxaban prevent thrombosis by directly inhibiting the activity of clotting factor Xa to prevent platelet activation and cross-binding of clotting proteins (56). When used with anticoagulant drugs, VFP complements their mechanisms, jointly preventing venous thrombosis and more effectively reducing the incidence of VTE than anticoagulant drugs alone. Therefore, the combination of VFP and anticoagulant drugs may effectively reduce postoperative VTE in orthopedic surgery patients (57), with VFP serving as a necessary complement to drug prevention.

### 4.3 Comparison between VFP and LMWH

Meta-analysis results show no significant difference in the effectiveness of VFP and LMWH in preventing postoperative VTE in orthopedic surgery patients. Although VFP and LMWH may operate through different mechanisms, their ultimate goal is to reduce blood stasis and promote blood circulation. LMWH reduces thrombus formation by inhibiting coagulation factor activity and increasing the activity of anticoagulant protein C (58). Conversely, VFP promotes blood flow through movement and pressure application, preventing venous blood stasis (59). Due to these similar mechanisms, VFP and LMWH are comparably effective in preventing postoperative VTE. Therefore, VFP can be considered as an alternative strategy to LMWH in patients at high risk of bleeding.

### 4.4 Comparison of effects between different usage times of VFP intervention

Subgroup analysis revealed non-significant differences in the effects of different VFP usage times, whether combined with anticoagulant drugs or not. This may be linked to the small number of studies with varied VFP usage durations included in this analysis. Among these, the frequencies of “30 min BID” and “24 h” were represented in about six studies, while other VFP durations were included in only one to two studies. Moreover, individual patient differences, implementation of the intervention, and duration may contribute to variability in the results, affecting their statistical significance.

## 5 Limitations

The scope and variety of VFP intervention times included in this study were not comprehensive, and the impact of the VFP intervention duration was not thoroughly examined. Additionally, the quality of the included literature was mostly moderate. Therefore, a more comprehensive multicenter, large sample, high-quality studies of VFP intervention with varying usage durations are needed to verify these findings.

## 6 Conclusions

Based on the current evidence, VFP can effectively prevent the occurrence of postoperative VTE in patients undergoing major orthopedic surgery. Combining VFP with drugs provides even better prevention. VFP can be considered an add-on strategy to LMWH for patients at low risk of bleeding and an alternative strategy to LMWH in patients at high risk of bleeding. Subgroup analysis revealed a non-significant difference in the effectiveness of VFP interventions with different usage durations. Future studies should conduct more comprehensive comparative research on VFP intervention with varying use times to help decision-makers choose the most effective treatment duration with limited resources, providing reference and practical guidance for thromboprophylaxis.

## Data Availability

The original contributions presented in the study are included in the article/Supplementary Material, further inquiries can be directed to the corresponding author.
